# The Principles and Practice of Modern Surgery

**Published:** 1844-10

**Authors:** 


Art. VIII.—*The Principles and Practice of Modern Surgery.* By Robert Druitt, Surgeon. From the third London edition. Illustrated with 153 wood engravings. With notes and comments: by Joshua B. Flint, M.D., M.M., S.S., late Professor of Surgery in the Medical Institute of Louisville. Philadelphia: Lea & Blanchard. 1844. 8vo. pp. 568.

The American editor of Druitt's '*Vade Mecum*' does not believe, that '*a rose by any other name would smell as sweet.*' He very shrewdly guessed, that the modest, and, it must be confessed, somewhat indefinite title of *vade mecum*, would not take so well with his title-loving countrymen, as the loftier one of 'The Principles and Practice of Modern Surgery.' He, therefore, wisely doffed the former and imposed the latter; and this is by far the most important service that he has rendered to the volume of Mr. Druitt. His 'notes and comments' are few and short; and if they had been even fewer and shorter than they are, neither 'Modern Surgery' nor its readers would have lost much. Prolixity in a *vade mecum* would be an unpardonable fault, and it would not have entered into the mind of many to append notes to such a production; but, we will own, it is probable that fewer still would have found it in their hearts, after beginning the task of commentator, to stop so soon. The notes are generally of a harmless character, and in some, the editor brings forward his own cases in a way to exhibit himself advantageously to the reader. Upon one of the longest of them we feel disposed to make a remark or two. To the chapter on *diseases of the rectum and anus* he has appended the following speculations:

"Nothing has been more remarkable, in my surgical experience in the west, than the disproportioned frequency of diseases of the rectum and adjacent textures—fistula, piles, prolapsus, &c., and I advert to the fact chiefly for the purpose of adding a cautioning remark respecting the causes of it. Doubtless it is partly to be referred to the chafing and contusions incident to horse-back riding which is a much more common mode of traveling here, than at the east; but it is mainly attributable to the habit of indiscriminate and excessive purgation so prevalent both as a remedial and prophylatic measure.

"A large portion of the practitioners of the Valley of the Mississippi have been educated under a system of medicine whose theory regards portal congestion and hepatic derangement as the essential elements of all diseases, and whose practice consists, almost exclusively, in the exhibition of drastic purgatives.

"It is natural that the people should imitate the therapeutics of their medical advisers, when so simple and easily applied, and accordingly they are very much in the habit of drenching themselves, and teasing the alimentary canal on every occasion of illness, with some concentrated purgative, in the form of pills.

"Under one of the most constant laws of irritation in mucous canals, the terminating portions of the apparatus of defecation, is thus perpetually suffering under propagated as well as direct stimulation, and reacts in the various forms of disease under notice.

"Besides these direct mischiefs, and others involving the health in other ways, occasioned by the pernicious doctrines referred to—which are, indeed, themselves essentially empirical—they encourage the grossest species of quackery, by promoting the consumption of vast quantities of patent pills and other purgative nostrums.

"In proportion as a more rational pathology shall prevail among physicians, the habits of the population will undergo a corresponding change, and the preponderance of diseases of the rectum in the duties of the surgeon, may be expected to disappear accordingly."

The disproportioned frequency of stone in the bladder must, also, be a remarkable circumstance in the editor's surgical experience in the west. It can hardly have failed to strike him, that calculous affections are much more prevalent in Kentucky than in Boston. They, too, we suppose, are to be attributed 'to the habit of indiscriminate and excessive purgation,' which the editor asserts prevails with our practitioners and people. It may be that the editor has seen more *fever* in Louisville, than he was wont to encounter on the shores of the Atlantic. Are our autumnal fevers, likewise, to be traced to this 'excessive purgation?'

We have no doubt that purgatives have been abused by some practitioners, as emetics have been by many, as the lancet is by others, and as even the scalpel itself has been by a few; but to speak of the use of these valuable remedies by the physicians of the west as 'indiscriminate and excessive,' is to do great injustice to a body of practitioners of whom, without meaning to make invidious comparisons, we will say, that in their knowledge of western diseases, and their skill in treating the same, they are, at least, the equals of the American editor of Druitt's *Vade mecum.* Quinine is becoming the *succedaneum* for purgatives, in all this region where calomel has been so favorite a remedy. In a few years, no doubt, we shall have some medical philosopher ingeniously tracing all the maladies of the rectum, dyspepsia, stone, and fevers, to the 'indiscriminate and excessive' use of *quinine.*

The work of Mr. Druitt has taken well with the profession, as is shown by the fact that it has reached its third edition in London, and its second in this country. Whatever may be the judgment of the profession concerning the labors of the American editor, there can be but one opinion as to the value of the numerous wood-cuts with which the American publishers have illustrated the volume. The work aspires to no higher character than that of a *compend* of surgical science and practice. In that capacity—as a *vade mecum*—we presume it has been found convenient and profitable. Y.

